# Life Insurance

**Published:** 1871-10

**Authors:** Thos. K. Beecher


### THE BISTOURY.

VOL. VII. ELMIRA, N. Y., OCTOBER, 1871. NO. 3.

### Communications.

### LIFE INSURANCE.

THOS. K. BEECHER.

A friend of mine once insured his life for eight or ten thousand dollars. Not a great while afterward he was taken suddenly and severely ill, but after a few critical days rallied and recovered. He testified that he owed his recovery to his policy of insurance. "For," said he, "if I had had to go through those days knowing what I did about my business and how my family would be left, I should have worried so that I never could have got well.—And I tell you I believe in life insurance if for nothing else because it keeps a man from worrying when he is sick."

My friend was right. If head work be a burden to a man when he is well, much more must it be a hindrance to a sick man when he would fain get well. He who is taken sick should if possible discharge from his mind all thought, all care, and attain as nearly as may be a state of contentment without strong excitement of any kind. A man in this frame of mind, if he have in addition a good family physician and reasonably good nursing, is as well off as a sick man can be. And if for this tranquil, contented frame it be necessary that a man have a policy of life insurance, he cannot better invest his money than by insuring at once.

But in addition to the worry and anxieties about a man's wife and children, there is apt to come to him when very sick, anxiety about his "soul's salvation." Very sick people are therefore supposed to need in a peculiar manner the attention of ministers and priests.

I have often speculated as to the effect produced upon pious members of the Roman catholic church by receiving the sacrament and extreme unction, which is asked for and received by those only who are past help; hence the coming of the priest may be looked upon as a sort of death-warrant.

In a less degree the visits of pious and praying friends to a sick bed, no matter how cheerfully they may be conducted, invariably suggest to the sick person that in the esteem of friends they are in a bad way and not likely to get well.

I often waver when I hear of a friend's sickness, and cannot decide whether to go to see him or not, merely because I am a minister, and if I go I carry with me a shadow of suggestion—that the man is "dangerous" and therefore it is time for the minister to come round. The neighbors infer the same thing. So long as they see the doctor visiting a house, they say a man is sick; but when the minister comes to visit, they say he is "dangerous"—cannot live.

To what extent then do we priests and pastors, by the impression which we inevitably make upon our patients, produce the very result which our visits are held to predict?

I know but one remedy for this unfortunate state of things, and that is to divorce religion from sickness and death and espouse her to health and a hearty life.

Is it sensible, is it manly, is it reasonably decent even, for people to lead careless, prayerless, irreligious lives, until they are cornered by sickness and have no chance for choice, and then, when their body is feeble, their head unsteady, and all their powers failing, stimulated and damaged by superstitious fear, undertake to do in their last hour an act which, when properly done, exercises the whole man,—body, soul, and spirit?

If a policy of life insurance be found eminently tranquilizing and salutary when a man is sick, would not a policy of salvation, in other words, a well ordered and vigorous religious faith, be found equally serviceable? Has a man really a fair chance for getting well until he has lost his fear of death?

I have met physicians who asked me not to visit this or that patient on occount of the bad effect it would have upon their elasticity and hopefulness to see a minister; and I think these physicians were right. For I cannot believe that the gift of faith and immortal hope can be genuine and valuable, if its receiving taxes a man's waning strength so that he who would otherwise get well, dies because of his over-exertion in understanding and receiving salvation.

People are often urged to "get religion" as a preparation for death. But what I now urge is, that men get religion as a help to keep from dying. In order to meet sickness with the best chance of recovery, it seems to me in a high degree desirable that men should adjust themselves to a well ordered, manly scheme of religion or irreligion. If they are going to make use of priests, preachers and churches at all, do it while hearty. Or, on the other hand, if they decide that such helps are beneath the dignity of manly strength, that they declare the same and die as they live, without care or thought, prayer or comfort.

I make this appeal equally in the interest of men whom I long to respect, and of religion, of which I am an ordained minister. I dislike, I reject entirely the too common notion that religion is peculiarly important for sick people and the dying. I suspect that the exhibition of it when a man is in danger as often sinks him as saves him.

For the same reason, then, that it is unwise for a man about to die to fret about his wood-pile and insist that he must chop a cord of wood so as to keep his wife comfortable; or fret about his business, and insist that he must, with failing faculties, rally and waste his little remnant of strength, so as to save his children from beggary; and by these untimely efforts bring about the catastrophe that he fears;—for that same reason I would protest against a man's exhausting himself about his soul's salvation when he has not strength enough to keep his body alive.

Religion received by the sick for the first time is usually a sick religion. The religion that will stand a man in good stead in the hour when heart and flesh fail, is the religion that he sought and found when he was strong and hearty and possessor of all his faculties.—Such a man when sick or dying it is good to visit, for he takes no harm and he confers immense strength upon every visitor.

In the interest, therefore, of good health and long life, and that all our readers may have the best chance to get well when they are sick, I counsel: Provide for your family by getting your life insured, and provide for yourself a rest for the soul in God. So doing, when sick you will worry or hurry about neither one nor the other. Ask your doctor if this be not good advice.

